# Melatonin Prevents Early but Not Delayed Ventricular Fibrillation in the Experimental Porcine Model of Acute Ischemia

**DOI:** 10.3390/ijms22010328

**Published:** 2020-12-30

**Authors:** Alena S. Tsvetkova, Olesya G. Bernikova, Natalya J. Mikhaleva, Darya S. Khramova, Alexey O. Ovechkin, Marina M. Demidova, Pyotr G. Platonov, Jan E. Azarov

**Affiliations:** 1 Department of Cardiac Physiology, Institute of Physiology, Komi Science Center, Ural Branch, Russian Academy of Sciences, 167000 Syktyvkar, Russia; bernikovaog@gmail.com (O.G.B.); nmichaleva@gmail.com (N.J.M.); alexlena@inbox.ru (A.O.O.); j.azarov@gmail.com (J.E.A.); 2 Department of Molecular Immunology and Biotechnology, Institute of Physiology, Komi Science Center, Ural Branch, Russian Academy of Sciences, 167000 Syktyvkar, Russia; dkhramova@gmail.com; 3 Department of Therapy, Institute of Medicine, Pitirim Sorokin Syktyvkar State University, 55 Starovskiist., 167001 Syktyvkar, Russia; 4 Department of Cardiology, Clinical Sciences, Lund University, 22185 Lund, Sweden; marina.m.demidova@gmail.com (M.M.D.); pyotr.platonov@med.lu.se (P.G.P.); 5 V.A. Almazov National Medical Research Center, 197341 Saint Petersburg, Russia; 6 Arrhythmia Clinic, Skåne University Hospital, 22185 Lund, Sweden; 7 Department of Biochemistry and Physiology, Institute of Medicine, Pitirim Sorokin Syktyvkar State University, 55 Starovskiist., 167001 Syktyvkar, Russia

**Keywords:** melatonin, ischemia, ventricular fibrillation, depolarization, repolarization, pig

## Abstract

Antiarrhythmic effects of melatonin have been demonstrated ex vivo and in rodent models, but its action in a clinically relevant large mammalian model remains largely unknown. Objectives of the present study were to evaluate electrophysiological and antiarrhythmic effects of melatonin in a porcine model of acute myocardial infarction. Myocardial ischemia was induced by 40-min coronary occlusion in 25 anesthetized pigs. After ischemia onset, 12 animals received melatonin (4 mg/kg). 48 intramyocardial electrograms were recorded from left ventricular wall and interventricular septum (IVS). In each lead, activation time (AT) and repolarization time (RT) were determined. During ischemia, ATs and dispersion of repolarization (DOR = RTmax − RTmin) increased reaching maximal values by 3–5 and 20–25 min, respectively. Ventricular fibrillation (VF) incidence demonstrated no relations to redox state markers and was associated with increased DOR and delayed ATs (specifically, in an IVS base, an area adjacent to the ischemic zone) (*p* = 0.031). Melatonin prevented AT increase in the IVS base, (*p* < 0.001) precluding development of early VF (1–5 min, *p* = 0.016). VF occurrence in the delayed phase (17–40 min) where DOR was maximal was not modified by melatonin. Thus, melatonin-related enhancement of activation prevented development of early VF in the myocardial infarction model.

## 1. Introduction

Ventricular fibrillation (VF) is a frequent life-threatening complication of myocardial ischemia, and search for effective antiarrhythmic agents to be applied in myocardial infarction patients remains an important challenge. Possible proarrhythmic effects of antiarrhythmic drugs continue to be a limitation hampering their use for arrhythmia prevention [1]. Melatonin, a natural pineal secretory product, has been demonstrated to confer versatile beneficial effects including cardioprotection [2]. In experimental studies, melatonin brought about antiarrhythmic effects [3,4,5,6,7,8,9] during ischemia/reperfusion. Also, it has been reported to be safe in humans even when administered in high doses [10]. These properties make melatonin a promising substance for further testing as a potential antiarrhythmic treatment in the setting of myocardial infarction.

Exact mechanisms of melatonin effects are not clear. Generally, it acts via receptor-mediated pathways and by its antioxidative properties [2]. The antiarrhythmic effects of melatonin were originally ascribed to improvement of the redox state [4,8]. On the other hand, our previous study has demonstrated that long-term melatonin treatment decreased ventricular tachycardia and/or ventricular fibrillation (VT/VF) incidence by enhancing myocardial activation, which was independent of the oxidative stress development [7]. The vast majority of data obtained thus far comes from isolated rodent heart ischemia-reperfusion models, where development of myocardial ischemia and related arrhythmogenesis is expected to be quite different from that in humans. Importantly, that the isolated preparations lack autonomic influences, which might affect arrhythmogenesis [11] and might be modified by melatonin via central mechanisms (for review see [12]). Data from in vivo experiments in large mammals having preserved hemodynamic load and autonomic innervation are needed to assess melatonin effects in a clinically relevant context.

In this work in a porcine myocardial infarction model, we aimed to explore the effects of melatonin on the myocardial electrophysiological parameters known to be related to reentrant arrhythmias (activation delay, duration and dispersion of repolarization), heart rate as a surrogate index for autonomic output, and their associations with oxidative stress parameters and VT/VF incidence during ischemia progression. 

## 2. Results

### 2.1. Effects of Ischemia

Coronary occlusion resulted in expected electrophysiological alterations in affected myocardial regions. Due to the coronary anatomy and location of occlusion (just distal to the first diagonal branch of the left anterior descending (LAD)coronary artery), the most pronounced effects were observed in the left ventricular (LV) apex, interventricular septum (IVS) apex and IVS middle portion (ischemic regions). The middle portion of the lateral LV wall and base of IVS were less affected (border regions), and no changes were observed in the base of the LV lateral wall (normal region). 

**Figure 1:**
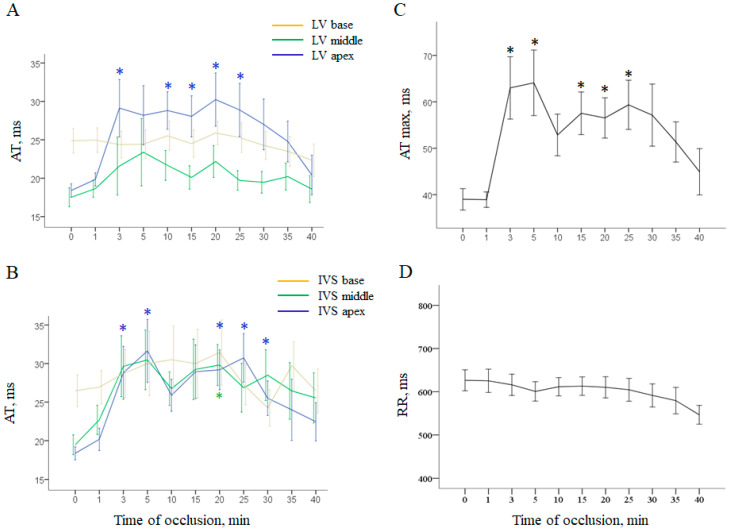
Ischemia-related changes of ventricular depolarization parameters (mean ± SEM). Panels (**A**,**B**) show local activation times (AT) in the left ventricular (LV) wall and interventricular septum (IVS), respectively. Panel (**C**) shows dynamics of maximal AT throughout all intramyocardial leads.RR dynamics are presented in panel (**D**). * denotes statistically significant differences in respect to baseline (0 min). See the most pronounced changes in the apical regions of the LV wall and IVS and less pronounced changes in the middle part of IVS. In the combined group (control + melatonin) basal regions do not demonstrate significant changes during occlusion. See a “spike and dome” pattern of maximal AT dynamics with two maxima at 3–5 and 15–30 min.

In the ischemic regions, mostly in the apex, activation times (ATs) abruptly increased as a manifestation of ischemia-related delay of activation spread (Figure 1). 

**Figure 2:**
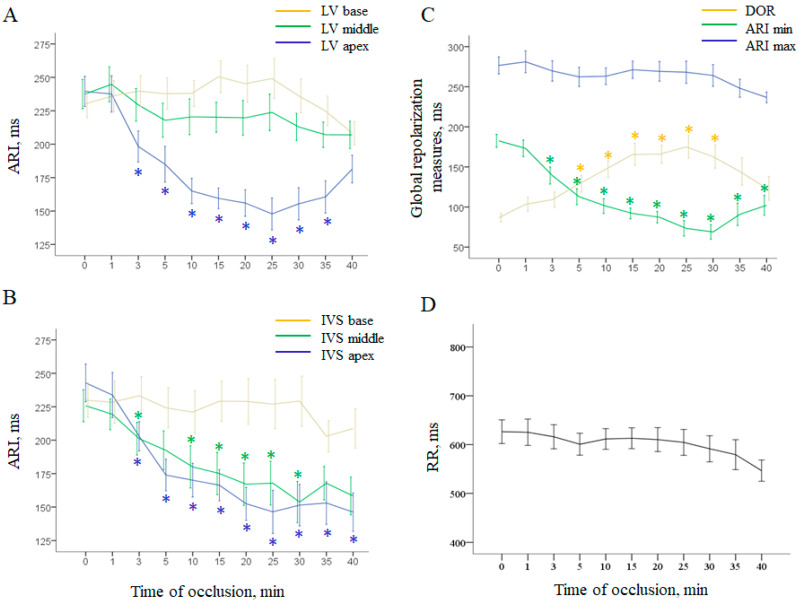
Ischemia-related changes of ventricular repolarization parameters (mean ± SEM). Panels (**A**,**B**) show local activation–repolarization intervals (ARI) in the left ventricular (LV) wall and interventricular septum (IVS), respectively. Panel (**C**) shows evolution of maximal and minimal ARIs throughout all intramyocardial leads and dispersion of repolarization (DOR). RR dynamics are presented in panel (**D**). * denotes statistically significant differences in respect to baseline (0 min). See distinct effects of coronary occlusion in the affected areas (LV and IVS apex and IVS middle part).

Repolarization measures changed more gradually (Figure 2). Activation-repolarization intervals (ARIs) shortened in the ischemic regions and stayed relatively stable in the normal and border regions (Figure 2A,B). As the minimal ARI duration characteristic of deeply affected ischemic regions decrease during occlusion, and the maximal ARI duration observed in the normal regions was unchanged, dispersion of repolarization (DOR) progressively increased (Figure 2C).

**Figure 3:**
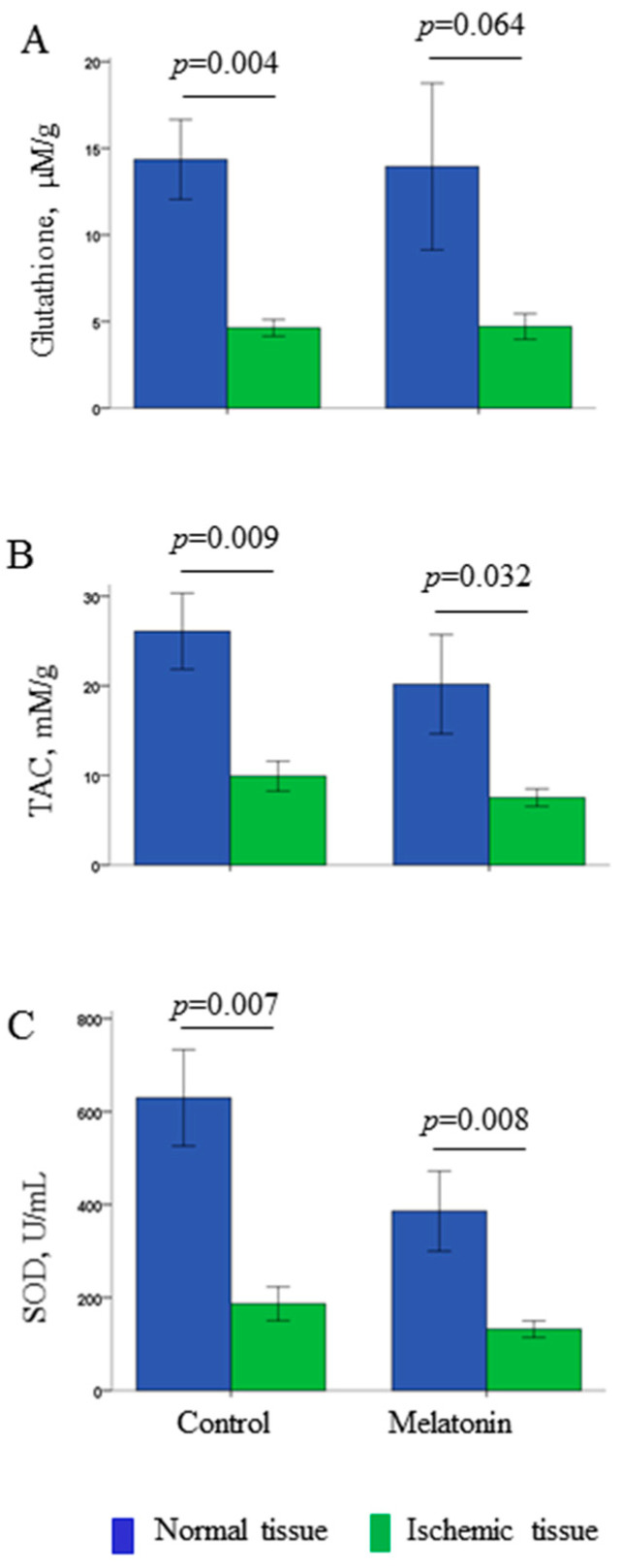
Interregional differences in the parameters (mean ± SEM) of redox state: total glutathione content (panel **A**), total antioxidant capacity (TAC, panel **B**), and superoxide dismutase activity (SOD, panel **C**). No differences between the control and melatonin-treated animals were found, and the ischemia-related changes were similar in the two groups.

Coronary occlusion also led to cardiac redox state modification. Compared with normal myocardium, all ischemic tissue samples were characterized by decreased levels of glutathione, total antioxidant capacity (TAC) and superoxide dismutase (SOD) activity (Figure 3).

### 2.2. Ventricular Fibrillation Incidence and Prediction

During acute myocardial ischemia, a total of 13 animals experienced VF. VF episodes clustered in early (1–5 min, five cases) and delayed (17–40 min, eight cases) phases, which are referred to as 1A and 1B phase, respectively. The studied electrophysiological parameters of ventricular depolarization and repolarization were tested as predictors of VF in a logistic regression analysis (Table 1). 

**Figure 4:**
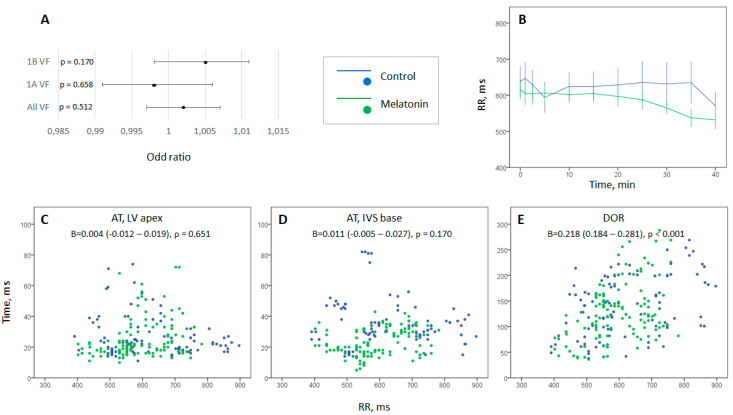
Heart rate dynamics and its contribution to arrhythmia-related parameters. Panel (**A**) shows odd ratio values with 95% CI and *p* values for association between RR duration and VT/VF occurrence. It demonstrates that VT/VF development was not predicted by heart rate changes. Panel (**B**) displays changes of heart rate (expressed as RR-interval duration, mean ± SEM) during coronary occlusion. No statistically significant differences were found neither between the control and melatonin groups, nor between baseline and any time-points during ischemia. Panels (**C**–**E**) display scatter plots for RR duration and major arrhythmia-associated myocardial electrophysiological parameters throughout all time-points: activation times in the ischemic zone (AT in the LV apex, panel **C**), activation times in the border zone (AT in the IVS base, panel **D**), and dispersion of repolarization (DOR, panel **E**). Regression coefficient with 95% CI and *p* values are pasted into the plots. See that RR duration was significantly associated with dispersion of repolarization (panel **E**), but not activation times (panels **C** and **D**); however, melatonin-treated animals demonstrate shorter ATs in the border zone irrespective of RR duration (panel **D**).

Among the studied parameters of local depolarization and repolarization, local ATs in the LV apex and IVS base, as well as maximal AT and DOR were significantly associated with VF incidence. Figure 4A shows that RR interval duration was not associated with neither early nor delayed VF development. The characteristics of oxidative stress were not associated with VF. 

### 2.3. Evaluation of Heart Rate Dynamics

Heart rate tended to increase during an episode of coronary occlusion, but this change was statistically insignificant in control and melatonin-treated animals, as well as in the combined (control + melatonin) group. No differences in heart rate dynamics were found between the groups (Figure 4B). The scatter plots of ventricular depolarization and repolarization parameters vs. RR-interval duration (Figure 4C–E) show AT both in ischemic and border zones were independent of heart rate, but dispersion of repolarization directly correlated with the RR-interval duration.

### 2.4. Melatonin Effects

**Figure 5:**
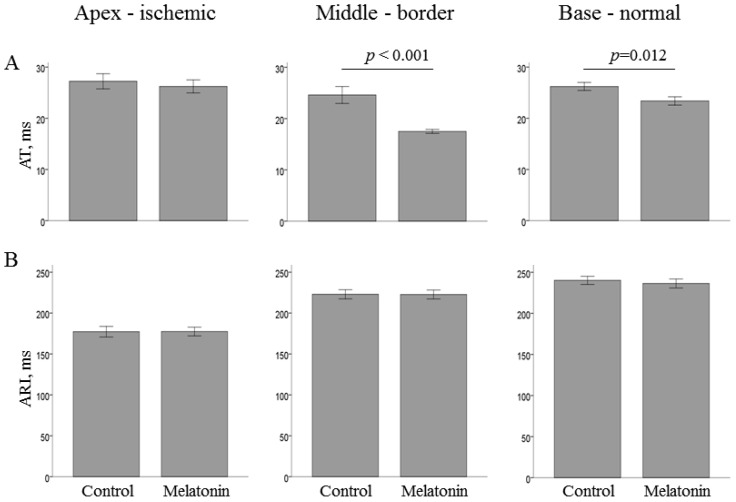
Effects of melatonin administration on local activation times (AT, panel **A**) and activation–repolarization intervals (ARI, panel **B**) in the ischemic (apex), border (middle part) and normal (base) regions of the left ventricle under coronary occlusion. Data are averaged over the period after 1 min from occlusion onset (time of melatonin infusion) until the end of ischemic exposure (mean ± SEM).

The studied parameters of oxidative stress in the normal and ischemic regions did not differ between the melatonin-treated and control groups (Figure 3). The average infarct size was nearly identical in the melatonin-treated (*n* = 5) and control (*n* = 4) animals that survived until the 40th min of occlusion (15.7 ± 8.3 vs. 15.7 ± 8.8%; *p* = 0.82, respectively). Figure 5 displays melatonin effects in the normal, border zone and ischemic myocardium of the LV wall.

ATs were shorter in the melatonin-treated animals in the basal and middle portions of the LV, areas that preserved, at least partially, perfusion during coronary occlusion episode. Overall, the most pronounced differences were found in the border regions, i.e., LV middle part (melatonin 17.5 ± 0.4 ms vs. control 24.6 ± 1.7 ms, *p* < 0.001) and IVS base (melatonin 23.0 ± 0.8 ms vs. control 35.4 ± 1.7 ms, *p* < 0.001). However, activation did not differ between the groups in the ischemic LV apex. Also, no differences concerning repolarization parameters were observed in any area.

**Figure 6:**
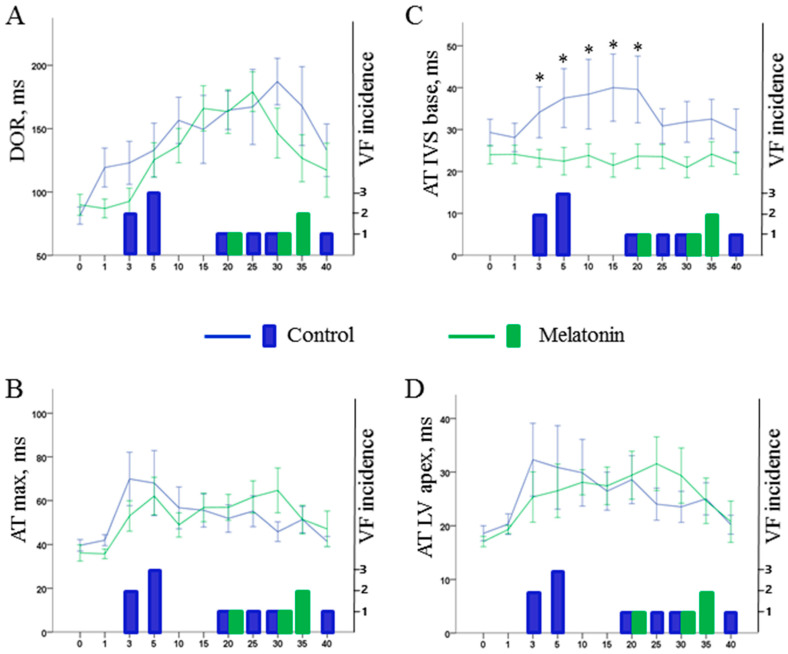
Dynamics of electrophysiological variables associated with ventricular fibrillation (VF) incidence during ischemic exposure, i.e., dispersion of repolarization (DOR, panel **A**), maximal activation time (AT max, panel **B**), local activation time in the base of interventricular septum (AT IVS base, panel **C**) and local activation time in the left ventricular apex (AT LV apex, panel **D**). Data are presented as mean ± SEM. Numbers of VF episodes at given time-points are presented in the lower part of each panel. See abrupt rise of ATs corresponding to the early cluster of VF and gradual increase of DOR with its maximum in the period of delayed VFs. Since pronounced ischemic changes were consistently observed in the LV apex, see similar time-courses of AT max and AT LV apex during coronary occlusion. * denotes statistically significant difference between control and melatonin-treated animals.

1A phase VF were absent in the melatonin-treated animals (0 out of 12 in melatonin group vs. 5 out of 13 in control groups, *p* = 0.016). However, 1B phase VF incidence was similar in the melatonin-treated (4 out of 12 animals) and control (4 out of 8 animals) groups (*p* = 0.456). Figure 6 shows the dynamics of the electrophysiological parameters associated with VF incidence in the logistic regression analysis (i.e., maximal AT, local AT in the LV apex and IVS base, and DOR) in the melatonin and control groups.

The only distinction between the control and melatonin-treated pigs concerned ATs in the IVS base. The difference developed shortly after occlusion onset and persisted until the 20th min. During this period, the control animals demonstrated significant activation delay, whereas the treated animals had their AT preserved at the preischemic level (Figure 6C).

## 3. Discussion

In this study, we observed acute effects of melatonin administered immediately after onset of coronary occlusion. No differences in the redox state parameters were observed between the treatment and control groups. The animals that received melatonin demonstrated shorter activation times in the perfused regions, which was associated with prevention of early (1A phase) VF. Although the effects on activation persisted, the delayed VFs (1B phase) were not prevented.

The absence of significant association between melatonin treatment, redox homeostasis and arrhythmias suggests that the observed antiarrhythmic effect of melatonin was realized via redox state-independent (implying receptor-mediated) pathway that involved electrophysiological targets. Antioxidative action is considered as one of the fundamentals of melatonin effects [2]. Melatonin was found to be effective for scavenging a variety of free radicals [13] and enhancing expression of antioxidant enzymes [14,15,16,17]. However, we did not observe any antioxidative action of melatonin in our study. The observation of the differences in the measured parameters of redox homeostasis between the normal and ischemic tissues in such conditions may be considered as a “quality control” implying that the applied method of assessment of oxidative stress was sensitive enough for detection of changes in the studied parameters. The probable explanation for the absence of melatonin effects may be that duration of the drug exposure was too short to produce any changes in the normal myocardium, while the ischemic areas were inaccessible for the treatment administered after occlusion. The infarct size caused by LAD occlusion was not modified by melatonin. VF episodes, especially those influenced by melatonin, occurred in several minutes after occlusion onset when necrosis development is not expected. The previous studies from other groups indicating importance of antioxidative action of melatonin [18,19,20,21] concerned an ischemia-reperfusion model where the drug could reach the affected tissue after reopening of the artery.

Activation of sympathetic system predisposes to ischemia-induced VF [22,23] that can be based on several mechanisms mediated by catecholamine-related [24] as well as neuropeptide Y-related [25] signaling. The proarrhythmic effects of sympathetic activation include facilitation of ectopic activity by abnormal automaticity and afterdepolarizations [26,27,28,29], increase in DOR [30] and inhomogeneous conduction [31]. Since intravenously infused melatonin can rapidly pass through the blood–brain barrier, it might be expected that it would influence electrophysiological properties via the sympatholytic action [12], which should have manifested in heart rate dynamics. However, no effects of melatonin on heart rate, and no association between heart rate and VF development were observed. The absence of these effects is possibly due to suppression of autonomic output in conditions of general anesthesia. The inhibitory effect of anesthesia can account for the absence of any relationship between heart rate and AT delay as well as cycle length-dependent increase (instead of expected decrease) of DOR (Figure 4). Collectively, these considerations suggest that the data obtained in the present experimental model demonstrate direct melatonin action on myocardial electrophysiological properties.

Enhancement of activation was the melatonin effect observed in the present study. It was found in the normal and border myocardium, i.e., the LV basal and middle part, and the IVS base. The fact that the activation parameters of ischemic regions did not differ between the groups supports association of the observed effects with the treatment since the substance administered in the bloodflow after induction of ischemia can reach only the regions with preserved perfusion.

The exact mechanism of activation improvement could not be elucidated in this study. Taking into account experimental settings, it can be suggested the AT changes resulted from respective changes in conduction velocity. There are number of factors causing conduction slowing in the ischemic myocardium. They include connexin function disturbances and depolarization of resting membrane potential leading to a decrease in sodium channel availability. We speculate that melatonin provided a salvage effect on conduction in the border zone. Theoretically, these preserving mechanisms may include upregulation of expression or phosphorylation of myocardial connexins [6,32,33]. However, it is unknown, if several minutes are sufficient for realization of this mechanism. Previously [7], we have shown that acute application of melatonin attenuates ischemia-reperfusion change of the resting potential, which can in turn influence availability of sodium channels and indirectly improve propagation. The latter explanation corresponds to our observation that the most pronounced effect of melatonin was observed not in the normal but border ischemic regions of the heart, where this “conduction sparing” effect was expected.

The melatonin antiarrhythmic effect in respect to 1A phase VF but not 1B phase VF might be due to a short half-life of melatonin. On the other hand, an electrophysiological explanation for the different effects on two arrhythmia phases can be presented as follows (see Figure 6). Generally, there are two major factors influencing VF development, namely activation delay and DOR. Activation delay developed rapidly after occlusion onset. The maximal AT delay had two distinct phases of increase manifesting in a “spike and dome” manner with the two phases of maximal AT delay corresponding to 1A and 1B phases of VF [34,35]. DOR increased gradually reaching its maximal values at 20–30 min of occlusion corresponding to 1B phase VF. Melatonin did not modify DOR but demonstrated positive effects in respect to activation. Though ATs in the ischemic regions (and maximal AT delay) could not be ameliorated due to inaccessibility of these regions, melatonin enhances activation in the perfused myocardium. Specifically, melatonin prevents significant AT delay in the border areas, namely IVS base and LV middle. Among two of them, AT delay in the IVS base was found to be associated with VF development in the present study as well as in our previous series of experiments with LAD occlusion in pigs [34]. The specific role of the IVS base in arrhythmogenesis cannot be explained directly by the findings of the present study. It might be related to its border localization in respect to the ischemic regions or to its late baseline activation (see time-point 0 in Figure 1B). Anyway, attenuation or aggravation of ischemia-induced conduction abnormalities in this area can decrease or increase the size of the affected core with impaired conduction which can be critical for formation of a reentrant circuit. Hence, melatonin improving activation spread in the critical for arrhythmogenesis region but having no effects in respect to repolarization prevents early but not delayed development of VF.

Study Limitations. General anesthesia inevitable for in vivo experiments imposed a limitation on the study due to its inhibitory effect on the sympathetic system. In the absence of such inhibition, both the arrhythmogenic response to ischemic insult and the antiarrhythmic effect of melatonin might have been more pronounced. Evaluation of redox state was limited to three parameters and infarct size estimation was done in a small number of animals. No parameters of autonomic balance and hemodynamical measures were studied save for heart rate. The electrophysiological effects of melatonin reported here concern the whole-organ scale, but molecular and ionic mechanisms were not directly addressed. These issues require cautious interpretations of the findings of the present study.

## 4. Materials and Methods

### 4.1. Animal Preparations and Experimental Protocol

The study conformed to the Guide for the Care and Use of Laboratory Animals, 8th Edition published by the National Academies Press (USA) 2011, the guidelines from Directive 2010/63/EU of the European Parliament on the protection of animals used for scientific purposesand was approved by the ethical committee of the Institute of Physiology of the Komi Science Centre, Ural Branch of Russian Academy of Sciences.

**Figure 7:**
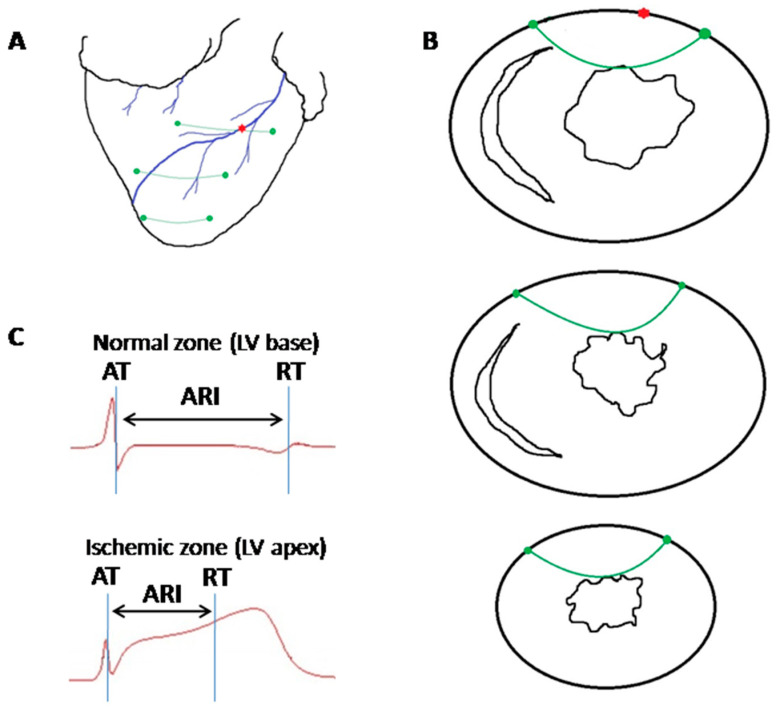
Electrophysiological recordings from the pig heart. Panels (**A**,**B**) show schematic presentation of the distribution of flexible plunge electrodes (green) in respect to superficial anatomical landmarks (panel **A**) and ventricular cross-cuts (panel **B**). Green filled circles indicate entry and exit points of electrode filaments. (**A**) red asterisk indicates the place of LAD ligation. Panel (**C**) shows representative electrograms with activation time (AT), end of repolarization time (RT) and activation–repolarization interval (ARI) markers. See significant ARI shortening in the electrogram from the ischemic zone (LV apex).

The experiments were performed in 25 pigs (30–45 kg body weight, both sexes). A porcine myocardial infarction model used in this study was described earlier [34]. The animals were anesthetized with zoletil (ZOLETIL^®^ 100, Virbac S.A., Carros, France, 10–15 mg/kg, i.m.), xylazine (Interchemie, Castenray, Netherlands, 0.5 mg/kg, i.m.) and propofol (Norbrook Laboratories Ltd., Newry, Northern Ireland, UK, 1 mg/kg, i.v.), intubated and mechanically ventilated. The heart was accessed via a midsternal incision. A ligature was placed (not tightened first) around the LAD artery just distal to the first diagonal branch origin (Figure 7A).

Three flexible plunge electrodes (16 lead terminals each) were drawn transmurally through the anterior portion of the LV, IVS and right ventricle at the apical, middle and basal levels (Figure 7A,B). Positions of electrodes were selected to comprise both ischemic and nonischemic areas. After the electrode and ligature placement and before a preischemic (baseline) recording, the heart was allowed to stabilize for 30 min. Then, the ligature was tightened, and the animals in the melatonin group (*n* = 12) were given melatonin (Sigma-Aldrich, St. Louis, MO, USA 4 mg/kg, intravenously) during the first minute of ischemia. Control animals (*n* = 13) received saline in the amount matching the volume of fluid given to the animals in the intervention group. Coronary occlusion was maintained for 40 min, during which electrophysiological recordings were done.

After the experiment, animals were euthanized under deep anesthesia by the intravenous potassium chloride injection either at the end of ischemic episode or immediately after VF development. The hearts were quickly excised; myocardial samples were taken from the apical left ventricular region (ischemic zone) and the right ventricular wall (normal zone) for measuring of oxidative stress (redox state) parameters (see below).

### 4.2. Electrophysiological Data Recording and Processing

Recordings were done at baseline and at 1, 2.5, 5, 10, 15, 20, 25, 30, 35, 40 min of coronary occlusion by means of a custom-designed system (16 bits; bandwidth 0.05 to 1000 Hz; sampling rate 4000 Hz). Unipolar intramyocardial electrograms were recorded in parallel with standard 12-lead ECGs. Local activation time (AT) and end of repolarization time (RT) were measured in each intramyocardial lead from the QRS onset to the instants of a dV/dt minimum during QRS-complex and dV/dt maximum during T-wave, respectively (Figure 7C). Activation–repolarization interval (ARI) was taken as a difference between RT and AT. RR interval was measured in the same cycles as AT, RT and ARI. Dispersion of repolarization (DOR) was calculated as a difference between the latest and earliest RTs throughout all recorded myocardial leads. Maximal AT, maximal and minimal ARIs were taken as maximal or minimal AT or ARI values appropriately throughout all recorded myocardial leads. In order to evaluate electrophysiological mechanisms of melatonin effects, we assessed electrophysiological parameters potentially related to arrhythmogenesis, such as average ATs and ARIs in the LV apex, LV middle part, LV base, IVS apex, IVS middle part, IVS base (local parameters) and DOR, maximal AT, maximal and minimal ARIs (global parameters) as well as early (1A phase, 1–5 min of occlusion) and delayed (1B phase, 15–40 min of occlusion) VF incidence.

### 4.3. Oxidative Stress Assessment

Parameters of oxidative stress were determined by the observers blinded to the treatment (placebo, *n* = 10 or melatonin, *n* = 11). Tissue samples were taken from ischemic LV and non-ischemic RV myocardium (a total of two samples from a heart, one sample from each zone). Every test was repeated twice, and average values of two tests were used for statistical evaluation.

The samples were rapidly submerged in liquid nitrogen and stored at −80 °C until further analysis. They were defrosted at 4 °C, rinsed with a phosphate buffer solution (PBS, pH 7.4) to remove any red blood cells and clots and blotted up. Then the samples were dissected, weighed using a laboratory scale AG204 DeltaRange (Mettler Toledo, Switzerland) and digested with Collagenase, Type 2 (WorthingtonBiochemical Corp., Lakewood, NJ, USA, 6 mg of the enzyme per g of tissue) in calcium-free Krebs–Henseleit buffer at 37 °C for 2 h. The digested samples were washed with PBS, blotted up, weighed and diluted with cold PBS (1 mL per mg of tissue) and sonicated with an ultrasonic cell disruptor on ice. The samples were pressed trough the filter membrane and resulting homogenates centrifuged for 15 min at 10,000× *g*. The levels of glutathione, total antioxidant capacity (TAC), and superoxide dismutase (SOD) activity were immediately analyzed in homogenates supernatants according to the manufacture’s protocols and expressed per g of tissue. Glutathione Assay kit (Sigma-Aldrich, St. Louis, MO, USA), Superoxide Dismutase Activity Assay (Cell Biolabs, San Diego, CA, USA), and Total Antioxidant Capacity Assay Kit (Cell Biolabs, San Diego, CA, USA) were used for analysis.

### 4.4. Infarct Size Estimation

Infarct size estimation was performed in the animals survived until the 40th minute of coronary occlusion. The hearts were excised and placed for 1 h in the freezer (–20 °C). Then, 10-mm-thick slices were made in apex-to-base direction. Slices were incubated for 15 min in a 1% 2,3,5-triphenyltetrazolium chloride solution (Sigma-Aldrich, St. Louis, MO, USA) at 37 °C and fixed for 1 day in 10% formalin to refine contrast. The normal myocardium was stained in brick-red color, and the infarct zone remained pale. Then slices were photographed, and the size of the infarct zone was determined as a ratio of the area of the infarct zone to the total cut-off area in each slice using open-source Fiji ImageJ software (version, Fiji contributors, University of Wisconsin-Madison, Madison, WI, USA).

### 4.5. Statistical Analysis

Statistical analysis was performed with SPSS package (IBM SPSS Statistics 23, Armonk, North Castle, NY, USA). Data are presented as mean ± SEM. Parametric tests were used according to the Kolmogorov-Smirnov normality test. General linear model for repeated measurements with Bonferroni corrections was used for assessment of ischemia effects on electrophysiological parameters. Comparisons between control and melatonin groups of animals were done with Student’s *t*-test. Univariate logistic regression analysis was used to assess relationships between predictors and VT/VF incidence. The differences were considered significant at *p* < 0.05.

## 5. Conclusions

Our findings demonstrate that melatonin can ameliorate arrhythmogenic substrate in a clinically relevant experimental model of myocardial infarction. It can prevent VF by a novel, redox state-independent mechanism, namely enhancing activation spread. However, its positive action concerns only the early VF, which can hardly be prevented by any treatment in the natural course of progression of myocardial infarction. However, it leaves room for its preventive use in conditions when an ischemic episode is anticipated, such as percutaneous coronary interventions or cardiac surgery.

## Figures and Tables

**Table 1:** Association of VF incidence with activation and repolarization parameters (univariate logistic regression analysis).

Predictor (Activation)	OR (95% CI)	*p*	Predictor (Repolarization)	OR (95% CI)	*p*
AT LV apex	1.058 (1.024–1.092)	0.001	ARI LV apex	0.992 (0.981–1.003)	0.136
AT IVS apex	1.017 (0.972–1.065)	0.459	ARI IVS apex	0.999 (0.990–1.009)	0.911
AT LV middle	1.006 (0.961–1.053)	0.785	ARI LV middle	1.003 (0.993–1.013)	0.511
AT IVS middle	1.023 (0.986–1.063)	0.230	ARI IVS middle	1.001 (0.991–1.010)	0.910
AT LV base	1.012 (0.945–1.085)	0.727	ARI LV base	1.008 (0.998–1.018)	0.100
AT IVS base	1.037 (1.003–1.072)	0.031	ARI IVS base	1.005 (0.995–1.015)	0.309
AT max	1.028 (1.010–1.047)	0.003	DOR	1.015 (1.005–1.026)	0.015

## Data Availability

The data presented in this study are available on request from the corresponding author. The data are not publicly available due to policy of the Institute of Physiology of the Komi Science Centre, Ural Branch of Russian Academy of Sciences.

## Abbreviations

IVS	Interventricular septum
LV	Left ventricle
AT	Activation time
RT	Repolarization time
DOR	Dispersion of repolarization
VF	Ventricular fibrillation
LAD	Left anterior descending coronary artery
VT/VF	Ventricular tachycardia and/or ventricular fibrillation
TAC	Total antioxidant capacity
SOD	Superoxide dismutase activity

## References

[1] Camm A.J. (2017). Hopes and disappointments with antiarrhythmic drugs. Int. J. Cardiol..

[2] Reiter R.J., Tan D.X., Galano A. (2014). Melatonin: Exceeding expectations. Physiology.

[3] Benova T., Knezl V., Viczenczova C., Bacova B.S., Radosinska J., Tribulova N. (2015). Acute anti-fibrillating and defibrillating potential of atorvastatin, melatonin, eicosapentaenoicacid and docosahexaenoicacid demonstrated inisolated heart model. J. Physiol. Pharmacol. Off. J. Pol. Physiol. Soc..

[4] Diez E.R., Prados L.V., Carrion A., Ponce Z.A., Miatello R.M.A. (2009). novel electrophysiologic effect of melatonin on ischemia/reperfusion-induced arrhythmias in isolated rat hearts. J. Pineal Res..

[5] Diez E.R., Renna N.F., Prado N.J., Lembo C., PonceZumino A.Z., Vazquez-Prieto M., Miatello R.M. (2013). Melatonin, given at the time of reperfusion, prevents ventricular arrhythmias in isolated hearts from fructose-fed rats and spontaneously hypertensive rats. J. Pineal Res..

[6] Egan Benova T., Szeiffova Bacova B., Viczenczova C., Diez E., Barancik M., Tribulova N. (2016). Protection of cardiac cell-to-cell coupling attenuate myocardial remodeling and proarrhythmia induced by hypertension. Physiol. Res. Acad. Sci. Bohemoslov..

[7] Sedova K.A., Bernikova O.G., Cuprova J.I., Ivanova A.D., Kutaeva G.A., Pliss M.G., Lopatina E.V., Vaykshnorayte M.A., Diez E.R., Azarov J.E. (2019). Association between Antiarrhythmic, Electrophysiological, and Antioxidative Effects of Melatoninin Ischemia/Reperfusion. Int. J. Mol. Sci..

[8] Tan D.X., Manchester L.C., Reiter R.J., Qi W., Kim S.J., El-Sokkary G.H. (1998). Is chemia/reperfusion-induced arrhythmias in the isolated rat heart: Prevention by melatonin. J. Pineal Res..

[9] Vazan R., Pancza D., Beder I., Styk J. (2005). Ischemia-reperfusion injury-antiarrhythmic effect of melatonin associated with reduced recovering of contractility. Gen. Physiol. Biophys..

[10] Andersen L.P., Gogenur I., Rosenberg J., Reiter R.J. (2016). The Safety of Melatoninin Humans. Clin. Drug Investig..

[11] Shen M.J., Zipes D.P. (2014). Roleofthe Autonomic Nervous Systemin Modulating Cardiac Arrhythmias. Circ. Res..

[12] Paulis L., Simko F. (2007). Blood pressure modulation and cardiovascular protection by melatonin: Potential mechanisms behind. Physiol. Res. Acad. Sci. Bohemoslov..

[13] Galano A., Reiter R.J. (2018). Melatonin and its metabolites vs. oxidative stress: From individual actions to collective protection. J. Pineal Res..

[14] Fischer T.W., Kleszczyński K., Hardkop L.H., Kruse N., Zillikens D. (2013). Melatonin enhances antioxidative enzyme gene expression (CAT, GPx, SOD), prevents their UVR-induced depletion, and protects against the formation of DNA damage (8-hydroxy-2′-deoxyguanosine) in ex vivo human skin. J. Pineal Res..

[15] Pablos M.I., Reiter R.J., Ortiz G.G., Guerrero J.M., Agapito M.T., Chuang J.I., Sewerynek E. (1998). Rhythms of glutathione peroxidase and glutathione reductase in brain of chick and their inhibition by light. Neurochem. Int..

[16] Kleszczyński K., Zillikens D., Fischer T.W. (2016). Melatonin enhances mitochondrial ATP synthesis, reduces reactive oxygen species formation, and mediates translocation of the nuclear erythroid 2-related factor 2 resulting in activation of phase-2 antioxidant enzymes (γ-GCS, HO-1, NQO 1) in ultraviolet radiation-treated normal human epidermal keratinocytes (NHEK). J. Pineal Res..

[17] Feng J., Chen X., Liu R., Cao C., Zhang W., Zhao Y., Nie S. (2018). Melatonin protects against myocardial ischemia–reperfusion injury by elevating Sirtuin3 expression and manganese superoxide dismutase activity. Free Radic Res..

[18] Dobsak P., Siegelova J., Eicher J.C., Jancik J., Svacinova H., Vasku J., Kuchtickova S., Horky M., Wolf J.E. (2003). Melatonin protects against ischemia-reperfusion injury and inhibits apoptosis in isolated working rat heart. Pathophysiol. Off. J. Int. Soc. Pathophysiol..

[19] Lagneux C., Joyeux M., Demenge P., Ribuot C., Godin-Ribuot D. (2000). Protective effects of melatonin against ischemia-reperfusion injury in the isolated rat heart. Life Sci..

[20] Sahna E., Parlakpinar H., Turkoz Y., Acet A. (2005). Protective effects of melatonin on myocardial ischemia-reperfusion induced infarct size and oxidative changes. Physiol. Res. Acad. Sci. Bohemoslov..

[21] Salie R., Harper I., Cillie C., Genade S., Huisamen B., Moolman J., Lochner A. (2001). Melatonin protects against ischaemic-reperfusion myocardial damage. J. Mol. Cell. Cardiol..

[22] Schwartz P.J., Billman G.E., Stone H.L. (1984). Autonomic mechanisms in ventricular fibrillation induced by myocardial ischemia during exercise in dogs with healed myocardial infarction. An experimental preparation for sudden cardiac death. Circulation.

[23] Collins M.N., Billman G.E. (1989). Autonomic response to coronary occlusion in animals susceptible to ventricular fibrillation. Am. J. Physiol.

[24] Wilder C.D.E., Pavlaki N., Dursun T., Gyimah P., Caldwell-Dunn E., Ranieri A., Lewis H.R., Curtis M.J. (2018). Facilitation of ischaemia-induced ventricular fibrillation by catecholamines is mediated by β1 and β2 agonism in the rat heart in vitro. Br. J. Pharmacol..

[25] Kalla M., Hao G., Tapoulal N., Tomek J., Liu K., Woodward L., Dall’Armellina E., Banning A.P., Choudhury R.P., Neubauer S. (2020). The cardiac sympathetic co-transmitter neuropeptide Y is pro-arrhythmic following ST-elevation myocardial infarction despite beta-blockade. Eur. Heart J..

[26] Barbieri M., Varani K., Cerbai E., Guerra L., Li Q., Borea P.A., Mugelli A. (1994). Electrophysiological basis for the enhanced cardiac arrhythmogenic effect of isoprenaline in aged spontaneously hypertensive rats. J. Mol. Cell. Cardiol..

[27] Boutjdir M. (1991). Alpha1-adrenoceptor regulation of delayed afterdepolarizations and triggered activity in subendocardial Purkinje fibers surviving 1 day of myocardial infarction. J. Mol. Cell. Cardiol..

[28] Cerbai E., Barbieri M., Mugelli A. (1996). Occurrence and properties of the hyperpolarization-activated current If in ventricular myocytes from normotensive and hypertensive rats during aging. Circulation.

[29] Hoppe U.C., Jansen E., Südkamp M., Beuckelmann D.J. (1998). Hyperpolarization-activated inward current in ventricular myocytes from normal and failing human hearts. Circulation.

[30] Irie T., Yamakawa K., Hamon D., Nakamura K., Shivkumar K., Vaseghi M. (2017). Cardiac sympathetic innervation via middle cervical and stellate ganglia and antiarrhythmic mechanism of bilateral stellectomy. Am. J. Physiol. Heart Circ. Physiol..

[31] Ajijola O.A., Lux R.L., Khahera A., Kwon O., Aliotta E., Ennis D.B., Fishbein M.C., Ardell J.L., Shivkumar K. (2017). Sympathetic modulation of electrical activation in normal and infarcted myocardium: Implications for arrhythmogenesis. Am. J. Physiol. Heart Circ. Physiol..

[32] Benova T., Viczenczova C., Radosinska J., Bacova B., Knezl V., Dosenko V., Weismann P., Zeman M., Navarova J., Tribulova N. (2013). Melatonin attenuates hypertension-related proarrhythmic myocardial maladaptation of connexin-43 and propensity of the heart to lethal arrhythmias. Can. J. Physiol. Pharmacol..

[33] EganBenova T., Viczenczova C., SzeiffovaBacova B., Knezl V., Dosenko V., Rauchova H., Zeman M., Reiter R.J., Tribulova N. (2019). Obesity-associated alterations in cardiac connexin-43 and PKC signaling are attenuated by melatonin and omega-3 fatty acids in female rats. Mol. Cell. Biochem..

[34] Azarov J.E., Ovechkin A.O., Vaykshnorayte M.A., Demidova M.M., Platonov P.G. (2019). Prolongation of the Activation time in ischemic Myocardium is Associated with J-wave Generation in ecG and Ventricular fibrillation. Sci. Rep..

[35] Demidova M.M., Martin-Yebra A., vanderPals J., Koul S., Erlinge D., Laguna P., Martinez J.P., Platonov P.G. (2014). Transient and rapid QRS-widening associated with a J-wave pattern predicts impending ventricular fibrillation in experimental myocardial infarction. Heart Rhythm Off. J. Heart Rhythm Soc..

